# LiDAR-Camera Calibration Using Line Correspondences

**DOI:** 10.3390/s20216319

**Published:** 2020-11-05

**Authors:** Zixuan Bai, Guang Jiang, Ailing Xu

**Affiliations:** School of Telecommunications Engineering, Xidian University, Xi’an 710071, China; zxbai_1@stu.xidian.edu.cn (Z.B.); alxu@stu.xidian.edu.cn (A.X.)

**Keywords:** extrinsic calibration, LiDAR-Camera, line correspondence, infinity point

## Abstract

In this paper, we introduce a novel approach to estimate the extrinsic parameters between a LiDAR and a camera. Our method is based on line correspondences between the LiDAR point clouds and camera images. We solve the rotation matrix with 3D–2D infinity point pairs extracted from parallel lines. Then, the translation vector can be solved based on the point-on-line constraint. Different from other target-based methods, this method can be performed simply without preparing specific calibration objects because parallel lines are commonly presented in the environment. We validate our algorithm on both simulated and real data. Error analysis shows that our method can perform well in terms of robustness and accuracy.

## 1. Introduction

Nowadays, with the popularity of unmanned vehicles, the navigation problems inherent in mobile robots are garnering even greater attention, among which the localization or calibration between different sensors is one of the basic problems. To fully utilize the information from sensors and make them complementary, the combination of 3D and 2D sensors is a good choice. Thus, the hardware devices of those systems are usually based on cameras and Light Detection and Ranging (LiDAR) devices. Comparing the two sensors, a camera is cheap and portable, and it can obtain color information about the scene, but it needs to correspond to feature points during calculation, which will be time consuming and sensitive to light. LiDAR can get 3D points directly and has an effective distance of up to 200 m. In addition, LiDAR is suitable for low-textured scenes and some scenes under varying light conditions. However, the data are sparse and lack texture information. When using a combination of cameras and LiDAR, it is necessary to obtain transformation parameters between coordinate systems of the two kinds of sensors. Once the transformation parameters, i.e., the rotation matrix and translation vector are obtained, the two coordinate systems are aligned, and the correspondence between 3D points and the 2D image is established. The 3D point cloud obtained by the LiDAR can be fused with the 2D image obtained by the camera.

The existing target-based methods require users to prepare specially designed calibration targets such as chessboard [1], circular pattern [2], orthogonal trihedron [3], etc., which limits the practicality of these methods. Target-less methods break through this limitation. These kinds of methods can be roughly divided into several categories according to work principles: odometry-based, neural network-based, and feature-based. The odometry-based methods [4,5] require many continuously inputted data, and the neural network-based methods [6,7] need even more data to train networks, and may lack clear geometric constraints. The feature-based methods usually use point or line features from scenes. Point feature is sensitive to noise, sometimes requiring user intervention to establish 3D–2D point constraints [8]. Line feature is more stable, and 3D–2D line correspondence is usually required (known as the Perspective-n-Line problem) [9,10,11]. However, in an outdoor environment, this correspondence is usually hard to be established. Because LiDARs are generally placed horizontally, many detected 2D lines on the image cannot find their paired 3D counterparts due to the poor vertical resolution.

**Figure 1:**
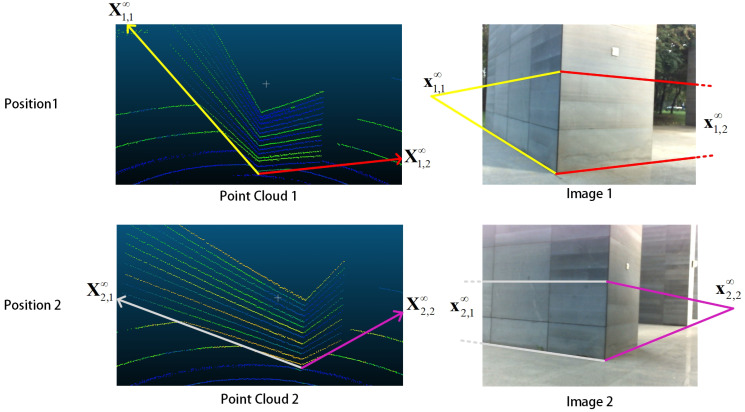
General constraints of our method. The first row shows the point cloud (top-left) and image (top-right) captured at position 1, and the second row shows the case of position 2. $X_{j,i}^{\infty }$ represents the 3D infinity points in the LiDAR coordinate system. $x_{j,i}^{\infty }$ represents the corresponding 2D infinity points on the image plane. During the position change, the relative transformation of the two coordinate systems is fixed. In our method, an initial solution can be obtained from at least two positions.

The main contribution of this paper is that we provide a novel line-based method to solve the extrinsic parameters between a LIDAR and a camera. Different from existing line-based methods, we take infinity points into consideration to utilize 2D lines, so that the proposed method can work in outdoor environments with artificial buildings, as shown in Figure 1. As long as there are enough parallel line features in a scene, it can be chosen as calibration environment. In addition, our method only requires a small number of data to achieve sufficient results. We transform the correspondence of parallel lines into the correspondence between 3D and 2D infinity points. By getting and aligning the direction vectors from the infinity points, the rotation matrix can be solved independently in the case that the camera intrinsic matrix is known. Then, we use a linear method based on point-on-line constraint to solve the translation vector.

## 2. Related Work

The external calibration between two sensors is always discussed. According to the different forms of data collected by these two devices, researchers have been looking for appropriate methods to obtain conversion parameters between the two coordinate systems. In some methods, the target is a chessboard, which is a plane object. Zhang et al. [1] proposed a method based on observing a moving chessboard. After getting points-on-plane constraints from images and 2D laser data, a direct solution was established to minimize the algebraic error, while they still needed several poses of planar pattern. Huang and Barth [12] first used chessboard to calibrate a multi-layer LiDAR and vision system. Vasconcelos et al. [13] formulated the problem as a standard P3P problem between the LiDAR and plane points by scanning the chessboard lines. Their method is more accurate than Zhang’s. Geiger et al. [14] arranged multiple chessboards in space to obtain enough constraint equations from a single shot. Zhou et al. [15] employed three line-to-plane correspondences, and then solved this problem with the algebraic structure of the polynomial system. Afterwards, they put forward their method based on the 3D line and plane correspondences and reduced the minimal number of chessboard poses [16]. However, the boundaries of the chessboard should be determined. Chai et al. [17] used ArUco marker, which is similar to the chessboard pattern, combined with a cube to solve the problem as a PnP problem. Surabhi Verma et al. [18] used 3D point and plane correspondences and genetic algorithm to solve the extrinsic parameters. An et al. [19] combined chessboard pattern with calibration objects to provide more point correspondences. However, those methods require the checkerboard pattern.

When spatial information is taken into account, some methods based on special calibration objects are proposed. Li et al. [20] provided a right-angled triangular checkerboard as calibration object. By using the line features on the object, the parameters can be solved. Willis et al. [21] used a sequence of rectangular boxes to calibrate a 2D LiDAR and a camera. However, the settings for the devices are demanding. Kwak et al. [22] extracted line and point features which are located on the boundaries and centerline of a v-shaped target. Then, they obtained the extrinsic parameters by minimizing reprojection error. Naroditsky et al. [23] used line features of a black line on a white sheet of paper. Fremont et al. [2] designed a circular target. In the LiDAR coordinate system, they used 1D edge detection to determine the border of the target and fitted the circle center and plane normal, but the size of the target needs to be known. Gomez-Ojeda et al. [3] presented a method that relies on an orthogonal trihedron, which is based on the line-to-plane and point-to-plane constraints. Pusztai et al. [24] used boxes with known sizes to calibrate the extrinsic parameters between a LiDAR and camera. Dong et al. [25] presented a method based on plane-line constraints of a v-shaped target composed of two noncoplanar triangles with checkerboard inside. The extrinsic parameters can be determined from single observation. These methods have high requirements for customized artificial calibration objects, and this may make them hard to be popularly adopted.

Some methods explored calibration methods without using artificial targets. These methods usually start with basic geometric information in a natural scene. Forkuo and King provided a point-based method [26] and further improved it [27], but the feature points are obtained by corner detector, which is not suitable for depth sensors with low resolution. Scaramuzza et al. [8] provided a calibration method based on manually selecting corresponding points. However, too many manual inputs will cause the results to become unstable. Mirzaei et al. [28] presented a line to line method by extracting the straight line structure. This algorithm is used for calibrating the extrinsic parameters of a single camera with known 3D lines, but it gives inspiration to the follow-up methods. Moghadam et al. [9] used 3D–2D line segment correspondences and nonlinear least square optimization to establish the method. This method performs well in indoor scenes, but, in outdoor scenes, the number of reliable 3D lines may not be adequate because of the viewing angle, low resolution of depth sensors, etc. This may lead to situations where many detected 2D lines cannot find their corresponding 3D counterparts. Levinson et al. [29] presented a method based on analyzing the edges on images and 3D points. This method only considers boundaries without extracting other available geometric information, and 3D point features may not be stable. Tamas and Kato [30] designed a method based on aligning 3D and 2D regions. The regions in 2D and 3D are separated by different segmentation algorithms, which may lead to inaccurate alignments of segmented regions and affect result accuracy. Pandey et al. [31] used reflectivity of LiDAR points and gray-scale intensity value of image pixels to establish constraints. By maximizing Mutual Information (MI), the extrinsic parameters can be estimated. Xiao et al. [32] solved the calibration problem by analyzing the SURF descriptor error of the projection of laser points among different frames. This method needs to input the transformation relationship among a large amount of images in advance. Jiang et al. [33] provided an online calibration method using road lines. They assumed that there are three lines which can be detected by both the camera and LiDAR on the road. This method is more similar to the following odometry-based methods and is suitable for automatic driving platform.

There are also some works based on other aspects (e.g., odometry and network). Bileschi [34] designed an automatic method to associate video and LiDAR data on a moving vehicle, but the initial relative pose between the sensors is provided by an inertial measurement unit (IMU). Schneider et al. [35] presented a target-less method based on sensor odometry for calibration. After this, they further gave an end-to-end deep neural method to calculate the extrinsic parameters [6]. Taylor and Nieto [4] presented an approach for calibrating the extrinsic parameters among cameras, LiDARs, and inertial sensors based on motion. Gallego et al. [36] provided a tracking method based on event camera in high speed application environments, but this method requires special devices and is used in special circumstances. Park et al. [5] aligned the odometry of the LiDAR and camera to obtain a rough estimation of extrinsic parameters, and then refined the results jointly with time lag estimation. These odometry-based methods require continuous input to estimate sensor trajectory, which demands many data. Cumulative errors are still a problem for odometry-based methods. However, they can work in targetless environments and are able to calibrate the extrinsic parameters continuously. With the development of neural networks, several novel methods appear. Schneider et al. [6] offered RegNet, which is the first convolutional neural network to estimate extrinsic parameters between sensors. Iyer et al. [7] presented a self-supervised deep network named CalibNet. Considering the Riemannian geometry, Yuan et al. [37] recently designed RGGNet to estimate the offsets from initial parameters. Neural network-based methods need more data to train the networks, and the performance is closely related to the training data.

## 3. Method

Throughout this paper the LiDAR coordinate system is regarded as the world coordinate system. The translation relationship of one point X in the world coordinate system to the image point x is

**Figure 2:**
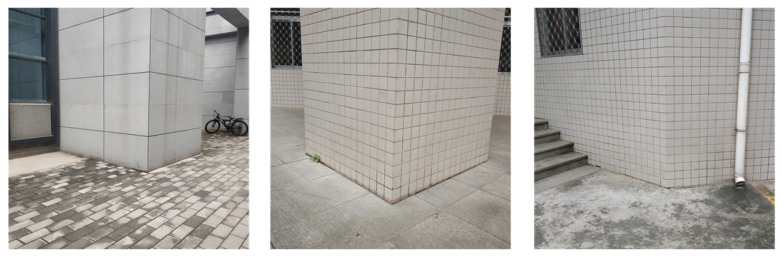
Some building corners in common scenes.

(1) x=K[R|t]X,

where K is the intrinsic matrix of the camera. It can be easily calibrated by traditional methods, e.g., Zhang’s method [38]. We aim to estimate the extrinsic parameters, i.e., rotation matrix R and translation vector t. To solve this problem, it is obvious that we need to find some features which can be detected in both the LiDAR point clouds and images. Considering robustness and commonality, line feature is an appropriate choice. In this paper, we choose the corners of common buildings to illustrate our method because they usually have sharp edges and available line textures, but this method can also be applied to any object with similar features. Some appropriate building corners are shown in Figure 2. We define each spin of LiDAR as a frame. We also define a frame and its corresponding image as one dataset. It is recommended to keep the devices fixed while collecting a dataset to avoid the distortion brought by movement.

### 3.1. Solve Rotation Matrix with Infinity Point Pairs

To solve rotation matrices, direction vector pairs are usually required. Many target-based methods use chessboards as the calibration object because it is convenient to get normal vectors of board planes in both the camera and the LiDAR coordinate systems. However, the board plane is small, and the LiDAR points on it are noisy, which makes it difficult to get sufficient results from a small number of data. Considering there are enough parallel lines in common scenes, we can obtain the vector pairs through the 3D–2D infinity point pairs based on line feature.

**Figure 3:**
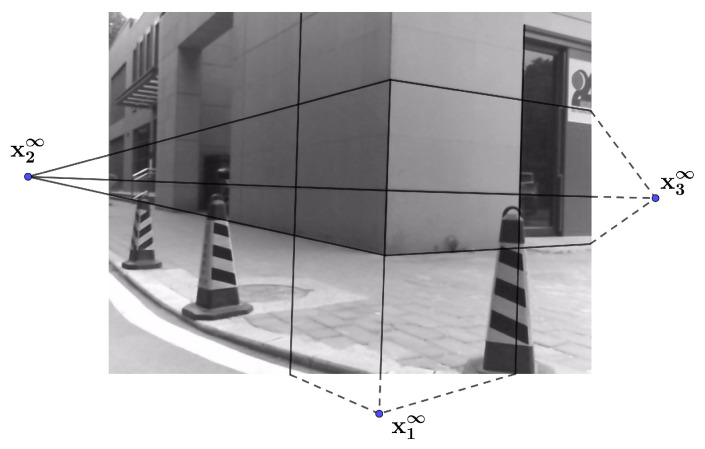
Corresponding points on an image plane.

One bunch of 3D parallel lines intersect at the same infinity point $X^{\infty }$, which lies on the infinite plane $\Pi^{\infty }$ in the space. Since 3D parallel lines are no longer parallel after perspective transformation, the intersection point of their projection lines is written as $x^{\infty }={\left[x_{1},x_{2},1\right]}^{T}$, which is not at infinity [39]. $X^{\infty }$ and $x^{\infty }$ make up a 3D–2D infinity point pair. Here, we use L-CNN [40] to detect the edges on an image. A RANSAC procedure is used to detect the infinity points of artificial buildings from images as in [41,42,43]. Then, three bunches of lines and their intersection points $x_{i}^{\infty },i=1,2,3$ on an image plane as shown in Figure 3 can be obtained. From Equation (2), we can get three 3D unit vectors $c_{1}$, $c_{2}$ and $c_{3}$.

(2) $$c_{i}=norm\left(K^{-1}x_{i}^{\infty }\right),\;i=1,2,3.$$

**Figure 4:**
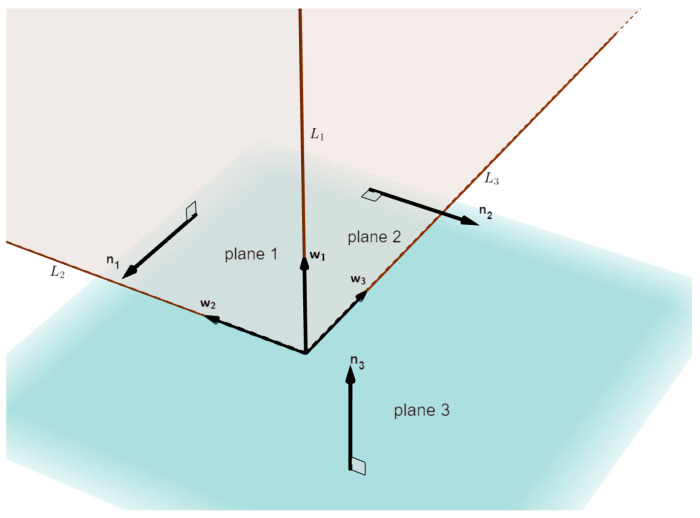
Geometric model in the LiDAR coordinate system.

When setting up the devices, an initial guess of the camera optical axis and LiDAR orientation can be obtained, i.e., a coarse relative pose of the LiDAR and camera is known. In the LiDAR point cloud, the planes of a building corner can be separated by existing point cloud segmentation methods [44,45,46]. The three planes shown in Figure 4 can be extracted according to the known orientation. The RANSAC algorithm [47] is used to fit the extracted planes. Then, we can get their normal vectors $n_{1}$, $n_{2}$ and $n_{3}$:

(3) $$\begin{array}{cc} A_{1}X+B_{1}Y+C_{1}Z+D_{1}=0,\; & n_{1}={\left[\begin{array}{ccc} A_{1}, & B_{1}, & C_{1} \end{array}\right]}^{T}\\ A_{2}X+B_{2}Y+C_{2}Z+D_{2}=0,\; & n_{2}={\left[\begin{array}{ccc} A_{2}, & B_{2}, & C_{2} \end{array}\right]}^{T}\\ A_{3}X+B_{3}Y+C_{3}Z+D_{3}=0,\; & n_{3}={\left[\begin{array}{ccc} A_{3}, & B_{3}, & C_{3} \end{array}\right]}^{T} \end{array}$$

$w_{1},w_{2}$, and $w_{3}$ are the normalized cross products of the plane normal vectors. They are the direction vectors of the 3D lines $L_{1}$, $L_{2}$, and $L_{3}$, as shown in Figure 4.

(4) $$\begin{array}{c} w_{1}=\pm norm\left(n_{1}\times n_{2}\right)\\ w_{2}=\pm norm\left(n_{1}\times n_{3}\right)\\ w_{3}=\pm norm\left(n_{3}\times n_{2}\right) \end{array}$$

**Figure 5:**
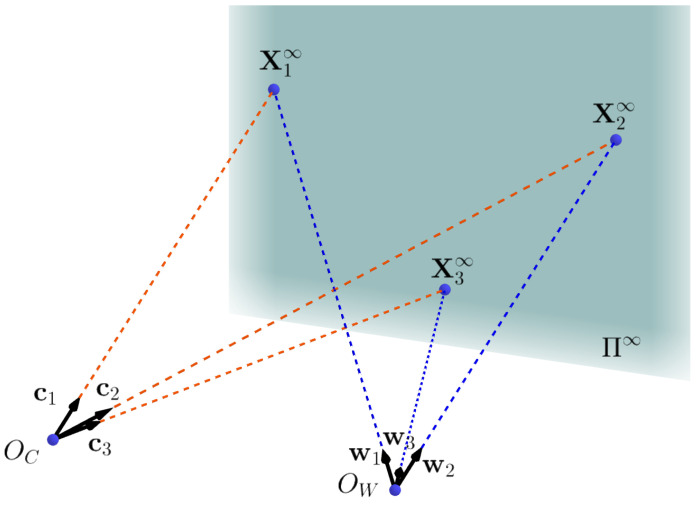
Geometric relationship between two coordinate systems. $O_{C}$ is the origin of the camera coordinate system. $O_{W}$ is the origin of LiDAR coordinate system. $\Pi^{\infty }$ is the infinity plane in the space. $X_{1}^{\infty }$, $X_{2}^{\infty }$, and $X_{3}^{\infty }$ are 3D infinity points in the world coordinate system. Actually, $c_{i}$ and $w_{i}$ coincide in the space.

Equation (5) shows the homogeneous form of the 3D infinity points in the LiDAR coordinate system. Notice that the direction (i.e., sign) of $w_{i}$ is still ambiguous, as shown in Equation (4). If $c_{i}$ is determined from an image, the direction it represents in the environment is roughly obtained. Then, its paired $w_{i}$ can be chosen by this condition, and the sign of $w_{i}$ can also be determined (consistent with the direction of $c_{i}$). Figure 5 shows the relationship between the two coordinate systems.

(5) $$\begin{array}{c} X_{1}^{\infty }=\left[\begin{array}{c} w_{1}\\ 0 \end{array}\right],X_{2}^{\infty }=\left[\begin{array}{c} w_{2}\\ 0 \end{array}\right],X_{3}^{\infty }=\left[\begin{array}{c} w_{3}\\ 0 \end{array}\right] \end{array}$$

Then, the direction vector pairs made up of $c_{i}$ and $w_{i}$ are obtained. The rotation matrix can be solved in close form with at least two pairs of direction vectors [48,49]. Assume there is a unit direction vector w in the world coordinate system. The relationship between w and its paired vector c is c=Rw. For the *i* th pair, we have $c_{i}=Rw_{i}$. Let

(6) $$A=\sum_{i=1}^{n}w_{i}c_{i}^{T}.$$

Applying singular value decomposition to A, we have $A={\mathrm{UDV}}^{T}$ and $R=VU^{T}$. The minimum number of *n* to determine R is 2.

Furthermore, if we have three or more vector pairs, R can also be solved in a simpler way:

(7) $$R=\left\{\begin{array}{cc} CW^{-1} & n=3\\ \left(CW^{T}\right){\left(WW^{T}\right)}^{-1} & n>3 \end{array}\right.$$

where $C=\left[\begin{array}{c} c_{1},c_{2},\cdots ,c_{n} \end{array}\right]$, $W=\left[\begin{array}{c} w_{1},w_{2},\cdots ,w_{n} \end{array}\right]$. The rank of matrix W must be greater or equal to 3 in this equation. Before computing R, the 3D infinity points should be checked to ensure that at least three directions in the space are selected. The solved R may not be orthogonal due to noise. To keep R as an orthogonal matrix, let $R=[r_{1},r_{2},r_{3}]$ be

(8) $$\begin{array}{c} \left\{\begin{array}{cc} r_{1} & =r_{1}/norm\left(r_{1}\right),\\ r_{2} & =r_{2}/norm\left(r_{2}\right),\\ r_{3} & =r_{1}\times r_{2}. \end{array}\right. \end{array}$$

### 3.2. Solve Translation Vector

The method presented above allows us to estimate rotation matrix R without considering translation vector t. Taking R as a known factor, here we use a linear method to get t.

**Figure 6:**
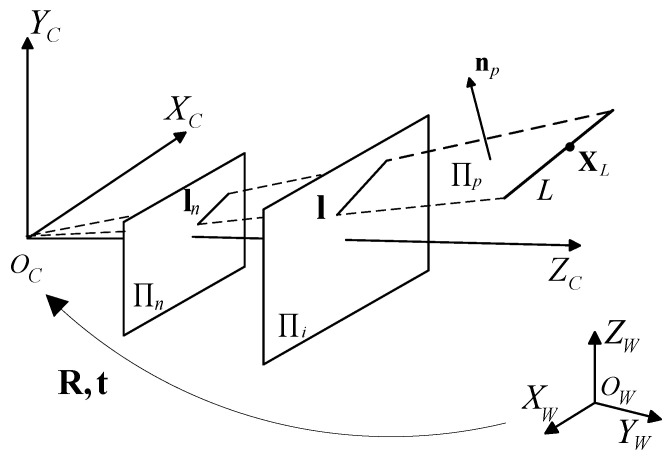
Geometry of camera projection model. $\Pi_{i}$ is the image plane,. $\Pi_{n}$ is the normalized image plane, and $\Pi_{p}$ is the interpretation plane.

Assume that there is a 3D point $X_{L}$ located on the line *L* in the LiDAR coordinate system as shown in Figure 6. $l_{n}:a_{n}x+b_{n}y+c_{n}=0$ and l:ax+by+c=0 are the projections of *L* on the normalized image plane $\Pi_{n}$ and image plane $\Pi_{i}$, respectively. $n_{p}={\left[a_{n},b_{n},c_{n}\right]}^{T}$ is the normal vector of the interpretation plane $\Pi_{p}$. We can obtain $n_{p}$ easily from l through the known intrinsic matrix of the camera. For each pair of corresponding lines, we have one equation for t [50]:

(9) $$n_{p}^{T}\left[R|t\right]X_{L}=0.$$

**Figure 7:**
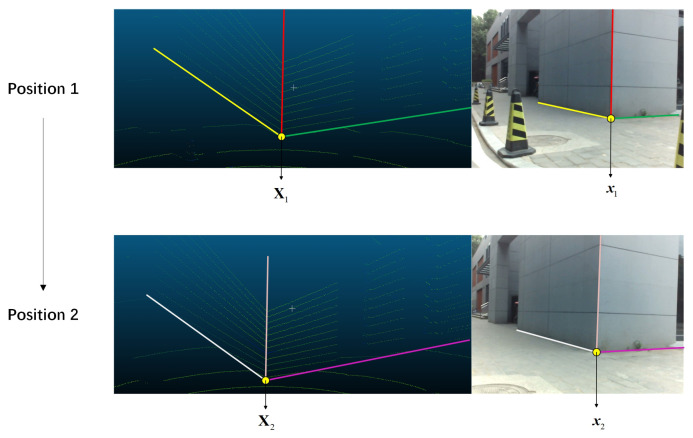
Two datasets from different positions. The lines marked with the same color correspond.

With the fitted planes in Equation (3), the 3D wall intersection lines $L_{1}$, $L_{2}$, and $L_{3}$ are easy to obtain, as is the 3D point $X_{L}$, which lies on the 3D line. We choose $L_{i}$ in the LiDAR coordinate system and $l_{i}$ on the image as corresponding line pairs. In general case, if three different sets of corresponding line pairs are known, the translation vector t can be solved. However, in our scene, $l_{1}$, $l_{2}$, and $l_{3}$ intersect at the same point on the image plane. This leads to a case that the three equations based on line constraint are not independent [51]. Thus, the equation factor matrix cannot be full rank, and this makes it difficult to solve t from a single dataset. To avoid this, we choose to move the LiDAR and camera and use at least two datasets to compute t. An example is shown in Figure 7, and then we can choose any three disjoint lines from these data to solve the problem.

### 3.3. Optimization

To make the results more accurate, the minimum of R and t under some constraints needs to be found. In this part, we construct cost function and then minimize the reprojection error to optimize R and t. We use the intersection of lines to establish the constraint. Assume that we have collected n(n≥2) datasets. For each set, we can get the intersection points x and X of the 2D and 3D lines, as shown in Figure 7. The cost function is:

(10) $$\begin{array}{c} \begin{array}{c} min\sum_{i=1}^{n}∥x_{i}-K\left[R|t\right]X_{i}∥. \end{array} \end{array}$$

We solve it by nonlinear optimization methods, such as the Levenberg–Marquard (LM) algorithm [52]. For the initial solutions with very low accuracy, we regard them as outliers and reject them before optimizing. The filter procedure is based on the RANSAC algorithm; a distance threshold for the reprojection error is set to distinguish the initial solutions. In this way, we can solve R and t and remove the influence from noise as much as possible. The complete process for the algorithm is described in Algorithm 1.
**Algorithm 1:**
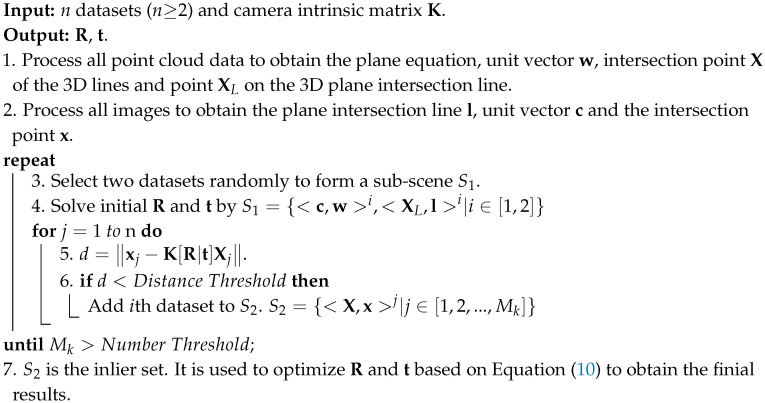


Similar to Fremont’s work [2], we estimate the precision of the calibration solution by the Student’s *t*-distribution. The covariance matrix $C_{ov}$ of the estimated parameters is defined as follows:

(11) $$C_{ov}=\epsilon^{2}\cdot {\left(J^{T}J\right)}^{-1}$$

where $\epsilon^{2}$ is an unbiased estimate of the variance and J is the Jacobian matrix of the last LM algorithm iteration. Then, the width of confidence interval is given by:

(12) $$\delta_{C_{i}}=t_{value}\cdot \sqrt{C_{ov}\left(i,i\right)}$$

where $\sqrt{C_{ov}\left(i,i\right)}$ is the standard deviation of the *i*th parameter. $t_{value}$ is determined by the degrees of freedom of the Student’s *t*-distribution and confidence (e.g., 95%).

## 4. Experiments

We conducted two experiments to verify our method. The first one was set up from simulated data to prove the veracity of our method and evaluate its robustness to noise. The second one used real outdoor data collected by a Leishen C16 LiDAR Scanner and a stereo camera system. We regard the transformation between the two cameras as ground truth. By comparing it with our results, we can quantify the accuracy of our method in real environments.

### 4.1. Simulated Data

**Figure 8:**
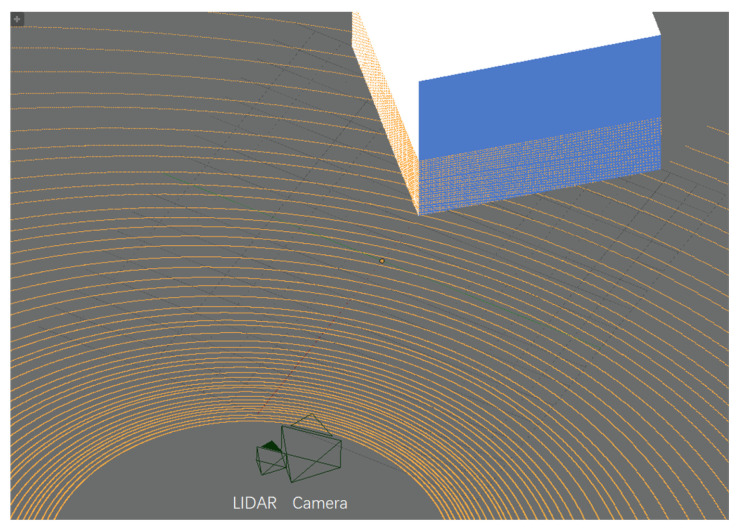
Simulation scene in Blensor. The yellow lines are LiDAR scan lines.

We used Blensor [53] to create simulated data. It is a sensor simulation package with different kinds of devices. In this experiment, we set Velodyne HDL-64E as the LiDAR, which works at 24 Hz with an angle resolution of $0.17^{\circ }$. The resolution of the virtual camera was set to 1920×1080 pixels, and its focal length was 30 mm. The intrinsic parameters are shown in Table 1. We established a scene and set up a virtual LiDAR-Camera pair to collect data, as shown in Figure 8.

**Figure 9:**
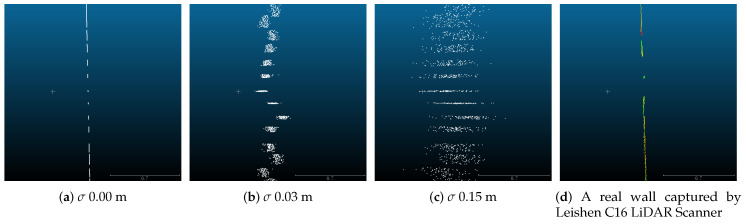
Side view of a wall to show noise effects on noise on point clouds. The measurement scale of the four maps is the same. The ruler at the right bottom of the images is measured in meters. The scan points on planes becomes quite noisy when σ becomes closer to 0.15 m.

The translation vector between the virtual LiDAR and camera was set to $\hat{t}={\left[-1,0,0\right]}^{T}$ in meters; the rotation matrix was $\hat{R}=\left[\begin{array}{ccc} 1 & 0 & 0\\ 0 & -1 & 0\\ 0 & 0 & -1 \end{array}\right]$; and Gaussian noise was added to the LiDAR point cloud to verify our method and test the robustness. The standard deviation σ was set to vary from 0.00 to 0.15 m. Figure 9 shows the effects from noise to point clouds. For each level of noise, we collected 10 datasets from different poses by moving the LiDAR-Camera pair. We first randomly chose 2 datasets to compute an initial solution and used the other 8 sets to optimize. We then repeated this procedure 100 times. The average of the 100 results was regarded as the extrinsic transformation under this noise level. Then, we calculated the error of rotation and translation for all noise levels:

(13) $$\begin{array}{c} e_{R}=\left|\theta_{R}-\theta_{\hat{R}}\right|\\ e_{t}=\left|t-\hat{t}\right| \end{array}$$

where [R|t] is the estimated transformation and $\left[\hat{R}|\hat{t}\right]$ is the ground truth. $\theta ={\left[\theta_{x-axis},\theta_{y-axis},\theta_{z-axis}\right]}^{T}$ is the Euler angle form of the rotation matrix.

**Figure 10:**
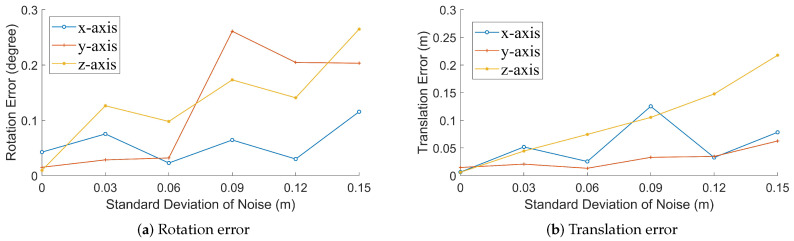
Errors for simulated data with different noise level.

Figure 10 shows the rotation and translation errors in our method. With the increase in noise, errors also increase. However, the rotation error of a single axis does not exceed $0.3^{\circ }$, and the translation error is still lower than 30 cm when σ=0.15 m. This shows that our method can provide a stable and accurate solution.

### 4.2. Real Data

In this experiment, we utilized a stereo camera system and a Leishen C16 LiDAR Scanner to capture point clouds. Generally, our method does not require a second camera. To evaluate our method quantitatively, we utilized a stereo camera system with pre-calibrated extrinsic parameters between the two cameras. The proposed method can calibrate the extrinsic parameters between a LiDAR and a single camera. When using a stereo camera system, we can calibrate two pairs of extrinsic parameters between the LiDAR and two cameras separately, and then we can estimate the extrinsic parameters between the two cameras from them. We regarded the pre-calibrated parameters as ground truth. By comparing the estimated results and the ground truth, we could analyze the accuracy of our method. A comparison with Pandey’s method [31] is also given.

**Figure 11:**
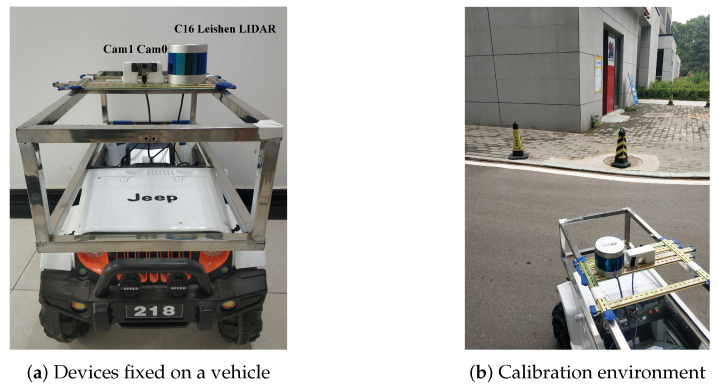
(**a**) Stereo camera system and C16 Leishen LiDAR Scanner. The two cameras provide ground truth parameters. (**b**) The environment of calibration.

The LiDAR works at 10 Hz with an angle resolution of $0.18^{\circ }$. The two cameras have a resolution of 640×480, and the relative pose between the LiDAR and stereo camera system is fixed. Figure 11a shows the devices, while Figure 11b shows the scene. The stereo camera system was pre-calibrated through Zhang’s method, and the intrinsic parameters are shown in Table 2. In the mean time, the extrinsic parameters were also determined, as shown in Table 3.

In the calibration scene, we placed the vehicle in front of a building corner and moved it in any direction 25 times. Each pose provided dataset. From any 2 of them, we could get initial extrinsic parameters $R_{Lto0}$ and $t_{Lto0}$ ($R_{Lto1}$ and $t_{Lto1}$) from LiDAR to Cam0 (Cam1). Then, we could get the extrinsic parameters $R_{0to1}$ and $t_{0to1}$ from Cam0 to Cam1 through a simple transformation:

(14) $$\begin{array}{cc} \; & R_{0to1}=R_{Lto1}R_{Lto0}^{-1} \end{array}$$

(15) $$\begin{array}{cc} \; & t_{0to1}=t_{Lto1}-R_{0to1}t_{Lto0} \end{array}$$

**Figure 12:**
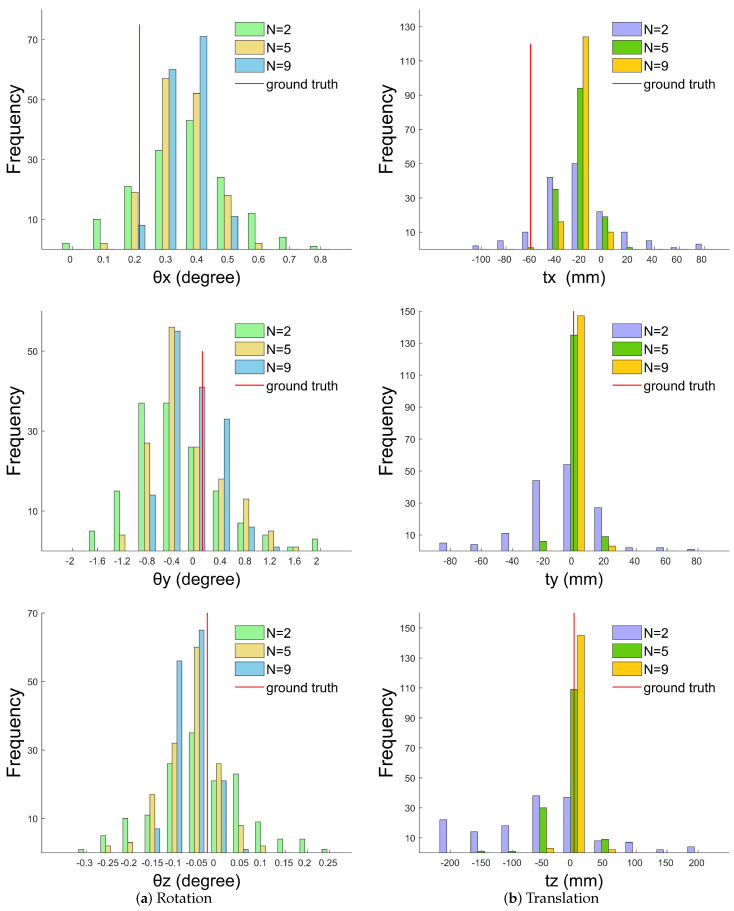
Distribution of initial extrinsic parameter solutions. *N* represents the number of poses we use to get an initial solution.

We randomly chose N(N>2) sets from among the 25 sets 150 times so that 150 initial extrinsic solutions from Cam0 to Cam1 were created. Let N=2,5,9; the distribution of initial extrinsic solutions is shown in Figure 12. It is clear that, with the increase of *N*, the data distribution gradually improved and tended to be stable. The red line in the graph represents ground truth. After comparing the initial solutions with the real value, it is clear that optimization is still required to refine the results.

**Figure 13:**
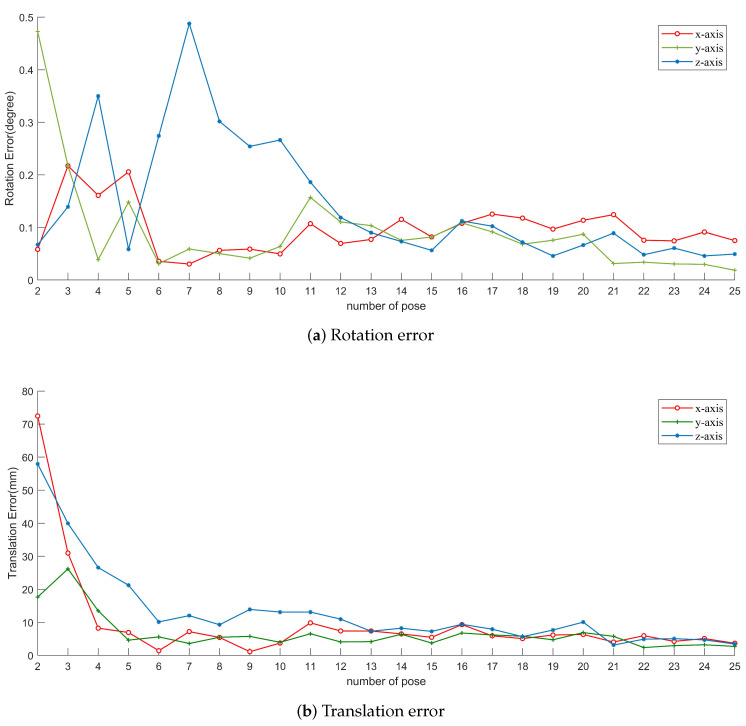
Errors for real data with respect to the number of poses we used.

The rotation and translation errors after optimization are shown in Figure 13. In the beginning, the errors are big and unstable. When more poses are used to optimize, the total error for the three axes decreases. After about 10 poses, the results become stable, and the translation error gradually decreases to below 1 cm. The result solved from 25 poses is shown in Table 4; the confidence interval is calculated with confidence 95%.

**Figure 14:**
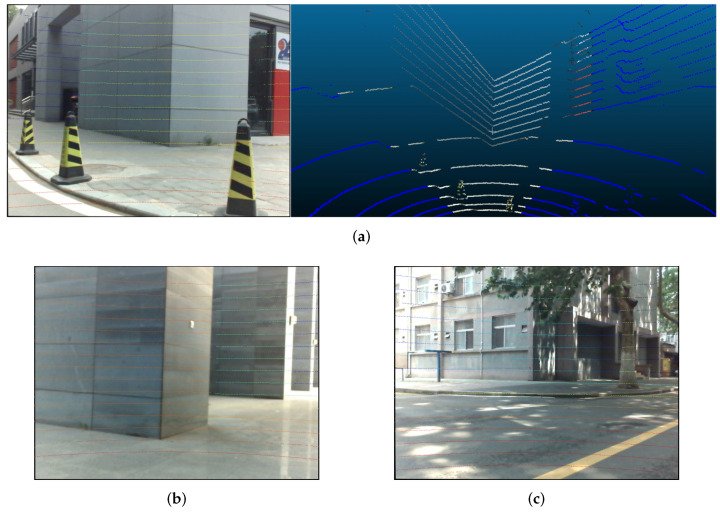
Visualized results: (**a**) a calibration scene, where the left part shows the point projection on the image captured by Cam0 and the right part shows the corresponding point cloud with extracted color information from the image; and (**b**,**c**) the projection result of other scenes using the same extrinsic parameters as (**a**).

**Figure 15:**
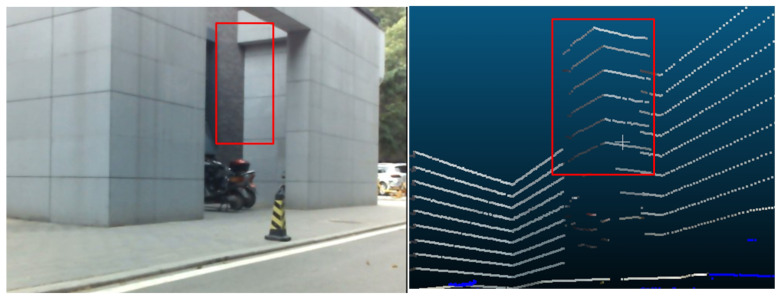
Detail of a colorized point cloud: the color of the wall changes correctly at the edge. The left and right part shows the image and the colorized point cloud, respectively.

The visualized results are shown in Figure 14. The colors of the projected points in Figure 14a,b are determined according to distance. The misalignments of some points (such as the blue and cyan ones on the traffic cone in Figure 14a) are caused by occlusion, because the points are observed by the LiDAR but not by the camera. In Figure 15, we can also intuitively observe the accuracy of the colorized results. The pose between the LiDAR and camera is well estimated.

To further show the performance, we compared Pandey’s method using the same 25 datasets. By calibrating the extrinsic parameters from the LiDAR to Cam0 and Cam1, respectively, the relative pose between Cam0 and Cam1 was estimated. The rotation and translation errors of Pandey’s method and ours are shown in Table 5. Our proposed method performs better due to the use of more geometric constraints of artificial buildings.

## 5. Conclusions

In this paper, we present a LiDAR-Camera extrinsic calibration method without preparing specific calibration object. We start with obtaining the 3D infinity points from the point cloud. Because there are sufficient parallel lines in the scene, we can obtain their corresponding 2D infinity points on the image. By obtaining the direction vectors from the 2D infinity points and aligning them to the 3D ones, we can solve the rotation matrix. Next, t can also be solved by point-on-line constraint linearly. Experiments show that our algorithm can calibrate the extrinsic parameters between camera and LiDAR with accuracy in common outdoor scenes. Meanwhile, the algorithm can also be applied to any scene with similar parallel line features.

## Figures and Tables

**Table 1:** Intrinsic parameters of virtual camera.

$f_{x}$	$f_{y}$	$c_{x}$	$c_{y}$
1800	1800	960	540

**Table 2:** Intrinsic parameters of stereo cameras.

	$f_{x}$	$f_{y}$	$c_{x}$	$c_{y}$
Cam0	759.377	759.791	352.516	237.499
Cam1	764.215	764.137	318.168	257.122

**Table 3:** Calibrated extrinsic parameters from Cam0 to Cam1.

$\theta_{x}$	$\theta_{y}$	$\theta_{z}$
$0.215^{\circ }$	$0.092^{\circ }$	$-0.026^{\circ }$
$t_{x}$	$t_{y}$	$t_{z}$
−60.088 mm	−0.300 mm	0.217 mm

**Table 4:** Calibration result obtained with 25 poses.

	LiDAR to Cam0	Confidence	LiDAR to Cam1	Confidence
Translation (m)				
$t_{x}$	−0.1041	±0.0078	−0.1622	±0.0077
$t_{y}$	−0.0324	±0.0093	−0.0297	±0.0087
$t_{z}$	−0.0211	±0.0154	−0.0177	±0.0145
Rotation (axis-angle)				
$r_{x}$	1.5549	±0.0019	1.5600	±0.0018
$r_{y}$	−0.0292	±0.0032	−0.0294	±0.0034
$r_{z}$	0.0495	±0.0037	0.0473	±0.0038

**Table 5:** Rotation and translation error (25 poses).

	$t_{x}$	$t_{y}$	$t_{z}$	$r_{x-\mathrm{axis}}$	$r_{y-\mathrm{axis}}$	$r_{z-\mathrm{axis}}$
Pandey [31]	0.0188 m	0.0305 m	0.0073 m	$0.4476^{\circ }$	$0.8375^{\circ }$	$0.4839^{\circ }$
proposed	0.0020 m	0.0028 m	0.0032 m	$0.0771^{\circ }$	$0.0138^{\circ }$	$0.0529^{\circ }$
